# Supplementary material for: Associations of Morphological Changes in Skeletal Muscles of Preschool Children in China Following Physical Activity
Source: Children (Basel). 2023 Sep 11;10(9):1538. doi: 10.3390/children10091538 (PMC10529834; doi:10.3390/children10091538)
Supplement: Supplementary file 1 [file children-10-01538-s001.zip › children-2572677-supplementary.pdf]

STROBE Statement

Table S1. Checklist of items that should be included in reports of ***cross- sectional studies.***

	Item No	Recommendation	Page in manuscript (subsection)
**Title and abstract**	1	(*a*) Indicate the study's design with a commonly used term in the title or the abstract	1 (title)
		(*b*) Provide in the abstract an informative and balanced summary of what was done and what was found	1 (abstract)
**Introduction**			
Background/ rationale	2	Explain the scientific background and rationale for the investigation being reported	2(background)
Objectives	3	State specific objectives, including any prespecified hypotheses	2 (last paragraph of background)
**Methods**			
Study design	4	Present key elements of study design early in the paper	2 (setting; participant recruitment)
Setting	5	Describe the setting, locations, and relevant dates, including periods of recruitment, exposure, follow-up, and data collection	2-3 (setting; participant recruitment)
Participants	6a	Give the eligibility criteria, and the sources and methods of selection of participants	2 (participant recruitment)
Variables	7	Clearly define all outcomes, exposures, predictors, potential confounders, and effect modifiers. Give diagnostic criteria, if applicable	2-3 (data collection and measures) 3-4 (data analysis);
Data sources/ measurement	8	For each variable of interest, give sources of data and details of methods of assessment (measurement). Describe comparability of assessment methods if there is more than one group	2-3 (data collection and measures)
Bias	9	Describe any efforts to address potential sources of bias	10 (limitations)
Study size	10	Explain how the study size was arrived at	Not applicable

Quantitative variables	11	Explain how quantitative variables were handled in the analyses. If applicable, describe which groupings were chosen and why	3-4 (data analysis);
Statistical methods	12	(*a*) Describe all statistical methods, including those used to control for confounding	3-4 (data analysis);
		(*b*) Describe any methods used to examine subgroups and interactions	3-4 (data analysis);
		(*c*) Explain how missing data were addressed	3-4 (data analysis);
		(*d*) If applicable, describe analytical methods taking account of sampling strategy	Not applicable
		(*e*) Describe any sensitivity analyses	Not applicable
**Results**			
Participants	13	(a) Report numbers of individuals at each stage of study— eg numbers potentially eligible, examined for eligibility, confirmed eligible, included in the study, completing follow-up, and analyzed	4 (general and socioeconomic characteristics)
		(b) Give reasons for non-participation at each stage	4 (general characteristics)
		(c) Consider use of a flow diagram	Not applicable
Descriptive data	14	(a) Give characteristics of study participants (eg demographic, clinical, social) and information on exposures and potential confounders	4(general characteristics); Table 1
		(b) Indicate number of participants with missing data for each variable of interest	Figure 1
Outcome data	15	Report numbers of outcome events or summary measures	4-6 (results)<br>Table 2-4<br>Figure 2
Main results	16	(*a*) Give unadjusted estimates and, if applicable, confounder-adjusted estimates and their precision (eg, 95% confidence interval). Make clear which confounders were adjusted for and why they were included	Not applicable
		(*b*) Report category boundaries when continuous variables were categorized	Not applicable
		(*c*) If relevant, consider translating estimates of relative risk into absolute risk for a meaningful time period	Not applicable

Other analyses	17	Report other analyses done— eg analyses of subgroups and interactions, and sensitivity analyses	Not applicable
**Discussion**			
Key results	18	Summaries key results with reference to study objectives	6 (first paragraph of discussion)
Limitations	19	Discuss limitations of the study, taking into account sources of potential bias or imprecision. Discuss both direction and magnitude of any potential bias	10 (limitations)
Interpretation	20	Give a cautious overall interpretation of results considering objectives, limitations, multiplicity of analyses, results from similar studies, and other relevant evidence	6-10 (discussion; limitations; conclusion)
Generalizability	21	Discuss the generalizability (external validity) of the study results	10 (limitations)
**Other information**			
Funding	22	Give the source of funding and the role of the funders for the present study and, if applicable, for the original study on which the present article is based	12 (competing interests)

Note: An Explanation and Elaboration article discusses each checklist item and gives methodological background and published examples of transparent reporting. The STROBE checklist is best used in conjunction with this article (freely available on the Web sites of PLoS Medicine at http://www.plosmedicine.org/(accessed on: 9 March 2000), Annals of Internal Medicine at http://www.annals.org/ (accessed on: 9 March 2000), and Epidemiology at http://www.epidem.com/(accessed on: 9 March 2000)). Information on the STROBE Initiative is available at www.strobe-statement.org (accessed on: 9 March 2000).
